# Mapping the Role of Robot-Assisted Gait Training in Post-Stroke Recovery Among Elderly Patients: A Scoping Review

**DOI:** 10.3390/jcm14113922

**Published:** 2025-06-03

**Authors:** Cinzia Marinaro, Lucia Muglia, Simona Squartecchia, Annalisa Cozza, Andrea Corsonello, Luigi Pranno, Maurizio Ferrarin, Tiziana Lencioni

**Affiliations:** 1 Istituto Nazionale di Riposo e Cura per Anziani (IRCCS INRCA), 87100 Cosenza, Italy; c.marinaro@inrca.it (C.M.); l.muglia@inrca.it (L.M.); or andrea.corsonello@unical.it (A.C.); l.pranno@inrca.it (L.P.); 2 IRCCS Fondazione Don Carlo Gnocchi, 20148 Milano, Italy; ssquartecchia@dongnocchi.it (S.S.); mferrarin@dongnocchi.it (M.F.); tlencioni@dongnocchi.it (T.L.); 3 Department of Pharmacy, Health and Nutritional Sciences, University of Calabria, 87036 Cosenza, Italy

**Keywords:** rehabilitation, elderly, stroke, robotics, gait disorders, scoping review

## Abstract

**Background/Objective:** Stroke is one of the leading causes of death and disability worldwide, with older survivors (aged > 65 years) bearing significant health and economic impacts, particularly in industrialized countries. While gait rehabilitation is a cornerstone in post-stroke recovery and robotic technologies offer promising tools to enhance its effectiveness, the existing literature has largely overlooked elderly populations. Most studies on robot-assisted gait training (RAGT)—which uses exoskeleton or end-effector devices to support and guide movement—either exclude older adults or do not analyze their outcomes separately. This review aims to critically evaluate the current evidence on RAGT in elderly post-stroke patients, addressing a significant gap in the literature and providing novel insights into the effectiveness and adaptability of RAGT for this specific population. **Methods:** The search included databases such as PubMed, Scopus, Embase, Web of Science, and ClinicalTrials. The inclusion criteria covered studies published up to March 2025, focusing on post-stroke individuals aged >65 years, who underwent RAGT. **Results:** 25 studies were included in the review, 21 involving exoskeleton and 4 end-effector devices. The primary focus was on motor outcomes, such as gait independence, gait parameters, and balance control. Only a few studies examined non-motor aspects, including cognitive and emotional functions, fatigue, pain, and neuroplasticity. Moreover, data on the long-term effects on the elderly population remain scarce. **Conclusions:** RAGT is an effective strategy for promoting motor recovery and improving functional outcomes, from independence in daily activities to quality of life, in the post-stroke elderly population. Early and high-intensity interventions are particularly useful with positive effects on neuronal plasticity, cognitive function, and well-being.

## 1. Introduction

Stroke is a major global neurological burden, ranking second among causes of mortality [1]. It is the third leading contributor to disability-adjusted life years (DALYs), integrating premature death and disability. In 2019, there were 12.2 million new stroke cases, causing 6.6 million deaths and 143 million DALYs [2,3]. The World Health Organization defines the elderly as people aged 65 and older, a population that currently accounts for 16.7% of the global population and is estimated to exceed 1.5 billion by 2050 [4,5]. This demographic trend, characterized by marked aging due to increased life expectancy and often referred to as a “demographic revolution”, suggests that elderly patients will represent the majority of people affected by stroke in the near future [6]. In the elderly, stroke impairs motor and cognitive domains, increasing mortality, prolonging hospital stays, and frequently requiring institutionalization. Aging alters stroke progression, with post-stroke motor disability leading to mobility deficits and increased fall risk [7,8]. At three months after stroke, 20% of individuals remain wheelchair-bound and 70% exhibit reduced walking speed [9].

Rehabilitation is the key to promoting motor recovery, particularly in elderly patients [10]. Modern technologies meet the demands of early, intensive, task-specific, and multisensory stimulation [11,12].

Robot-assisted gait training (RAGT) enhances neuromotor learning by promoting proper gait patterns. In the field of robotics and rehabilitation, end-effectors and exoskeletons represent two fundamental approaches for human-machine interaction: while end-effectors interact with the user externally and control movement through a single point of contact (e.g., feet for lower limb), exoskeletons integrate with the body, applying assistance or resistance directly across multiple joints to closely mimic natural human motion. Multiple studies and a recent meta-analysis have demonstrated the positive impact of robotic training in conjunction with physical therapy on functional, motor, and cognitive outcomes post-stroke, enhancing endurance and refining walking techniques [13,14,15,16]. A recent Cochrane review highlighted significant improvements in walking independence, particularly among non-ambulatory participants at the study’s outset and those receiving early intervention post-stroke [17].

Elderly post-stroke patients often face reduced functional autonomy due to multimorbidity, polypharmacy, nutritional deficits, and prolonged hospitalization, with cerebral events further worsening motor impairments [18]. Literature suggests that the effectiveness of RAGT in elderly post-stroke patients is promising but variable. Maranesi et al. [19] found that, although RAGT improves walking ability and independence in elderly patients, these results are not fully generalizable to the very elderly population, highlighting the need for targeted studies. Similarly, a retrospective case-control study by Manuli et al. [14] demonstrated that high-intensity robotic rehabilitation is feasible and well-tolerated in elderly stroke survivors; however, the variability in individual responses underlines the importance of patient stratification to optimize outcomes. Thus, while RAGT shows promise, its applicability and effectiveness in this population remain underexplored—probably partly due to lower adherence to evidence-based stroke care protocols with advancing age and the frequent exclusion of very elderly patients with comorbidities, which limits the generalizability of study findings [20]. This scoping review maps current knowledge on RAGT in post-stroke individuals over 65 years assessing gait and balance, non-motor outcomes, limitations, and future challenges.

## 2. Materials and Methods

### 2.1. Search Strategy

A scoping review approach following Preferred Reporting Items for Systematic Reviews and Meta-Analyses Extension for Scoping Reviews (PRISMA-ScR) guidelines was adopted to investigate the effectiveness and challenges of RAGT in elderly post-stroke patients [21]. This review was conducted without prior registration of the study protocol.

PubMed, Scopus, Embase, Web of Science, and ClinicalTrials electronic databases were searched for peer-reviewed and human studies. Given the differences in indexing and search functionalities across databases, tailored search strategies were applied to each platform. Search terms generally included combinations of “stroke”, “lower limb”, “gait”, and robotic-related keywords such as “robotic”, “exoskeleton”, “end-effector”, or “robot-assisted”. The exact search formula for each electronic database is included in Supplementary File S1.

Research was based on work published by March 2025, and was conducted from April to May 2025. Reference lists of relevant systematic reviews were also manually screened for additional eligible articles.

### 2.2. Eligibility Criteria

Eligibility criteria for study selection were divided into populations included in the studies, the concept involved, and the context in which the studies were conducted, following the PCC framework. Study designs were considered for inclusion in this review as follows:

Participants: We included studies that enrolled post-stroke patients aged ≥65 years, assigned either to the experimental or control group, with gait impairments requiring a RAGT-based rehabilitation intervention.

Concept: Studies assessing the use of robotic devices for the treatment of gait impairments, involving exoskeletal and/or end-effector devices, and evaluating motor and non-motor outcomes as either primary or secondary endpoints, were included.

Context: We included studies conducted in both inpatient and outpatient settings, during the acute, subacute, and chronic stages of stroke recovery. The post-stroke recovery phases were defined according to the Stroke Roundtable Consortium, as follows: the first 24 h as the hyperacute phase, 1–7 days as acute, 7 days–3 months as early sub-acute, 3–6 months as late sub-acute, and after 6 months on as chronic [22].

Study designs: Only primary studies (randomized controlled trials, retrospective studies, observational studies, case-control, cross-sectional, non-randomized controlled trials, and case reports) were considered. We included only articles published in English and excluded studies without full-text availability. Letters to the editor, opinion pieces, and reviews (including scoping and systematic reviews) were excluded; although, the reference lists of relevant reviews were screened for additional studies.

### 2.3. Study Identification

Two independent reviewers screened titles and abstracts according to predefined criteria, retrieving full-text studies for potentially eligible records and independently reviewing bibliographies. Any discrepancies were discussed and resolved through consensus. The initial consistency rate between the reviewers before the discussion was 80%. In cases of disagreement, a third reviewer was consulted. Duplicate records were identified and removed manually by comparing article titles. Eligible articles were evaluated by title, abstract, text, and scientific validity.

### 2.4. Data Extraction

**Figure 1:**
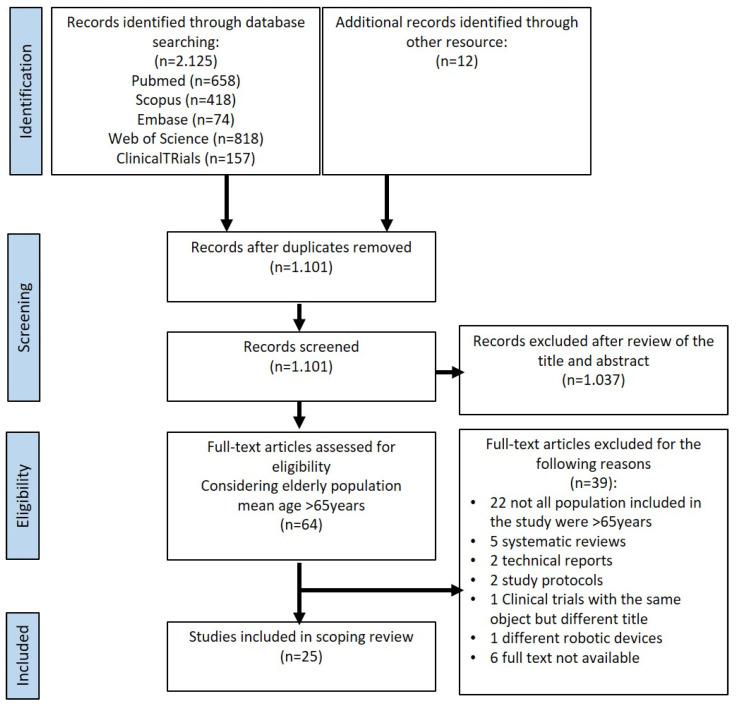
PRISMA Flowchart of the selection process.

For each eligible manuscript, reviewers extracted data using a predefined spreadsheet. The two reviewers identified data on motor and non-motor effects on post-stroke elderly patients using robotic devices for lower limb rehabilitation. Detailed information on data extraction is provided in the PRISMA flowchart (Figure 1). The analysis followed a narrative method to synthesize findings from different study designs and interventions.

### 2.5. Procedure for Effect Size Calculation

When available in the original studies, effect sizes (Cohen’s d) were directly extracted and reported in the results tables without modification. For studies that did not report effect sizes, these were calculated using the G*Power software (Heinrich Heine University), based on the statistical information provided in the articles. For within-group comparisons, means and standard deviations for pre- and post-intervention values were used. For between-group comparisons, calculations were performed using either pre-post mean differences (when explicitly provided) or pre- and post-intervention means and standard deviations, provided that baseline values were comparable between groups. In studies reporting only the median, this was used as an approximation of the mean. When the interquartile range (IQR) was reported, the standard deviation was estimated using the formula SD ≈ IQR/1.349, assuming a normal distribution. For results presented with 95% confidence intervals (CIs), the standard deviation was approximated using the formula SD ≈ (Upper limit − Lower limit of CI)/(2 × 1.96). Both within-group (pre-post, dependent means) and between-group (intervention vs. control, independent means) comparisons were considered for effect size estimation. Co-hen’s d values were interpreted as follows: small effect (d = 0.20–0.49), medium effect (d = 0.50–0.79), and large effect (d ≥ 0.80). 

## 3. Results

An overview of the PRISMA-ScR article selection process is provided in Figure 1. The original search resulted in a total of 2125 articles, additional records identified through other resources were 12. After the removal of duplicates, 1.101 articles were left for title and abstract screening. Based on the mean age threshold of ≥65 years of the study population, 64 articles were selected for full-text review. Finally, 25 studies met all inclusion criteria: n.14 Randomized Control Trial (RCT), n.1 retrospective study, n.1 retrospective observational study, n.1 retrospective cohort study, n.1 randomized, single-blind clinical trial, n.2 cross-sectional study, n.1 retrospective case-control study, n.1 non-randomized controlled trial, n.2 observational study, n.1 prospective open-label study were identified.

A detailed overview of the studies analyzed, considering the main motor outcomes reported, is provided in Table 1.

**Figure 2:**
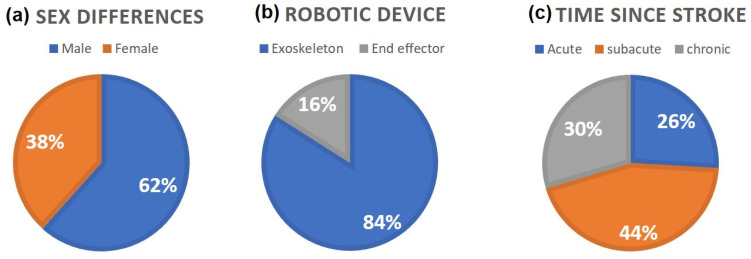
Illustration of study participant’s distribution based on (**a**) sex—62% male and 38% female, (**b**) the type of robotic device employed (exoskeleton vs. end-effector), and (**c**) time since stroke, categorized as acute, subacute, or chronic.

As stated in the eligibility criteria, we included only studies in which the mean age of post-stroke patients was ≥65 years in both the experimental and control groups. Across the included studies, the mean age of participants was approximately 69.8 years, with individual study means ranging from 65 to 78 years and substantial within-study variability, as reflected by standard deviations. For studies providing median and IQR data, reported median ages ranged from 55 to 71 years, with IQR generally extending from 55 to 75. Collectively, the findings confirm that the study populations were composed primarily of older adults, in line with the epidemiological profile of the target condition. The distribution of study participants by sex was: 62% male and 38% female (Figure 2a). Baseline Functional Ambulation Category (FAC) scores across the reviewed studies ranged from 0 to 3, reflecting varying degrees of functional ambulation, from complete dependence to supervised mobility.

### 3.1. RAGT and Stroke Recovery Timeline

According to the stroke recovery timeline defined by the Stroke Roundtable Consortium [22], the majority of the study participants were in the subacute phase (44%), while smaller proportions were in the acute (26%) and chronic (30%) phases (Figure 2c).

Studies investigating RAGT in acute stroke settings have shown positive effects on motor outcomes and quality of life, although no significant impact on balance has been reported [23,24,25,26,27,28]. The greatest benefits appear in patients with severe lower limb motor impairment, as indicated by lower baseline scores on the Fugl-Meyer Assessment for the Lower Extremity (FMA-LE) [27]. Notably, the use of exoskeleton devices such as the HAL system improved motor recovery during early post-stroke phases [25]. Moreover, early initiation of RAGT with exoskeletons was associated with a higher proportion of patients achieving supervised gait in a shorter timeframe, as well as a reduction in the length of stay in rehabilitation facilities [29].

Most studies focus on patients over 65 years in the sub-acute phase, when motor goals, like trunk control and partial walking independence, are achieved [30,31,32,33]. The goals most achieved at this stage involve control of static and dynamic balance [30,31,32,33], trunk-control and coordination [34], rhythmic neuromuscular pattern in the proximal lower limb muscles [35], walking independence [31,36], as well as cognitive functions [30,34].

In the chronic stage, RAGT improves motor, balance, quality of life (QoL), and emotional outcomes with sustained effects at 1- and 3-months post-treatment [14,34,37,38,39,40,41].

### 3.2. RAGT Devices

Considering the studies included, 21 studies employed exoskeleton devices [14,23,24,25,26,27,29,32,33,34,35,36,37,38,39,41,42,43,44,45,46], while only 4 used an end-effector [28,30,31,40] (Figure 2b).

**Table 1:** Overview of included studies. The study aims, design, sample size, device used, key interventions/methodology, and the main motor outcomes were reported.

Authors	Study Aims	Study Design	Sample Size	Device	Interventions and Methodology	Main Motor Results
Rha Y.H. et al., 2025 [41]	Compare the effectiveness of RAGT and traditional rehabilitation therapy on trunk symmetry and lower limb muscle strength in patients with chronic stroke.	Randomized, single-blind clinical trial	49 chronic stroke patients	Exoskeleton	Walkbot System (EG) vs. conventional therapy (CG) RAGT: 30 min/session, 3 times/week for 4 weeks. Measures: strength and stiffness of the paralyzed knee extensors, gait symmetry variables, trunk symmetry variables after 2 and 4 weeks.	Within group: in EG improvement in step length (d = 0.66) and muscle strength (d = 0.31). In CG improvement in step length (d = 0.83).
Kato D. et al., 2025 [29]	Investigate the effects of RAGT initiation within 1 week after onset on degree of gait independence in individuals with stroke.	Retrospective cohort study	36 post-stroke patients: 18 in acute and 18 in subacute phase	Exoskeleton	WellWalk device in acute and subacute post-stroke phase. RAGT: 40 min/session, 3–7 times/week Measures: FIM walk score; SIAS motor score; cumulative incidence of gait under supervision events respect to days from RAGT onset, days of RAGT, dose of rehab, actual gait time, gait distance.	Between group: In the acute group significantly higher percentage and faster achievement of gait under supervision (d^#^ = 2.62), with earlier RAGT initiation (d^#^ = 1.92). No differences in RAGT duration, rehabilitation time, gait training time, actual gait time, or gait distance.
Kubilius R. et al., 2024 [46]	Compare the differences in cardiac function, fatigue, and workload during ADLs wearing UAN.GO and OPTIGO walker in people with stroke.	pilot cross-sectional study	5 sub-acute stroke patients.	Exoskeleton	UAN.GO and OPTIGO walker uses in 3 experimental conditions (1: Walking w/o exoskeleton; 2: Walking with UAN.GO; 3: Walking with UAN.GO–OPTIGO platform). RAGT: 5 sessions in different conditions. Measures: HR and R–R interval of ECG data, SUS, TSQ-WT	Not reported
Kim Y. et al., 2023 [34]	Compare the effectiveness of HIT and conventional physiotherapy on cognitive and motor functions in patients with post-stroke dementia.	Retrospective clinical study	48 sub-acute-chronic patients with post-stroke dementia.	Exoskeleton	Walkbot-based human–robotic interactive gait training (EG) vs. conventional therapy (CG). RAGT: 60 min/session, 3 times/week for 6 weeks. Measures: MMSE, FMA, TIS, BBS, MBI.	Between group: in EG improvement in FMA compared to the CG (d = N.C.).
Lee Y.H. et al., 2023 [42]	Examine the effectiveness of robotic exoskeleton-assisted rehabilitation and identify predictive factors for significant improvement.	RCT	38 sub-acute stroke patients.	Exoskeleton	FREEwalk exoskeletal device (EG) vs. conventional therapy (CG). RAGT: 3 times/week robotic group, 2 times/week conventional group for 4 weeks. Measures: 6MWT, SF-12, isokinetic dynamometer.	Within group: in EG improvement in 6MWT (d^#^ = 0.99); peak torque force of the knee (flexion-extension) at 60/s (d flex^#^ = 0.62, d ext^#^ = 0.57) and 120/s (d flex^#^ = 0.39, d ext^#^ = 0.37).
Degami A. et al., 2023 [25]	Investigate whether early initiation of gait training using HAL improves functional outcomes in patients with stroke.	A Retrospective Observational Study	63 acute stroke patients.	Exoskeleton	HAL for Well-Being Lower Limb Type in early and late group of patients based on the days from stroke onset to initiation of gait training RAGT: 20 min/session, 3 times/week. For 25.59 ± 22.18 days in early group, 28.06 ± 26.14 in late group. Measures: BRS, mRS, and FIM	Between group: BRS of the lower limb was significantly higher in the early group than in the late group (d^#^ = 0.85).
Castelli L. et al., 2023 [30]	Evaluate the effects of rehabilitation with Hunova on cognitive function and balance in older adults with stroke.	RCT	24 sub-acute stroke patients.	End-Effector	Movendo Hunova robotic platform training (EG) vs. conventional therapy (CG). RAGT: 3 times/week for 4 weeks. Measures: FAB, SCWT, SDMT, DCT and TMT, BBS, SPPB. AI, WHS, FAC, mBI, EQ-5D, MFIS, FSMC, MI-LL.	Within group: in EG improvement in MI-LL (d^#^ = 2.22), AI (d^#^ = 0.77), WHS (d^#^ = 2.35). In CG improvement in MI-LL (d^#^ = 1.82), FAC (d^#^ = 1.01), WHS (d^#^ = 1.01). Between group: in EG improvement in MI-LL affected side compared to the CG (d^#^ = 0.59).
Firouzi M. et al., 2022 [43]	Evaluate the feasibility and effects of gait training with a novel wearable robotic device (HWA) for post-stroke rehabilitation.	Pilot study	5 sub-acute chronic stroke patients	Exoskeleton	Honda Walking Assist in normal walking, unassisted and optimal assisted conditions. RAGT: Single session. Measures: velocity (m/s); cadence (steps/min); paretic and non-paretic cycle time (s), paretic and non-paretic stride length (m), paretic and non-paretic stride velocity (m/s); paretic and non-paretic swing phase (% gait cycle); paretic and non-paretic stance phase (% gait cycle) and paretic and non-paretic double support phase (% gait cycle).	Within group: In EG with optimal assistance improve in spatiotemporal gait parameters: velocity (m/s) (d = 0.28), paretic and non-paretic stride length (m) (d paretic limb = 0.22, d non-paretic limb = 0.18).
Aprile I. et al., 2022 [31]	Evaluate the effectiveness of robotic gait and trunk rehabilitation compared to robotic gait training alone on balance, activities, and participation measures in patients with subacute stroke.	RCT	36 sub-acute stroke patients.	End-Effector	G-EO + Hunova Movendo (EG) vs. G-EO (CG). RAGT: 45 min/session, 3 times/week for 12 sessions/month. Measures: BBS score, MI-LL, MAS, ID Pain, NRS, mBI, AI, FAC, 10-MWT, 6 MWT, TCT, TIN-B, WHS.	Within group: in EG improvement in MI-LL (d^#^ = 0.65), MAS (d^#^ = 0.37), FAC (d^#^ = 0.51), WHS (d^#^ = 0.51), 6MWT (d^#^ = 0.52), AI (d^#^ = 2.70). In CG improvement in FAC (d^#^ = 0.78), AI (d^#^ = 0.75).
Manuli A. et al., 2021 [14]	Evaluate the feasibility and effectiveness of intensive robotic rehabilitation using the Lokomat Free-D in elderly patients.	Retrospective case-control study.	80 elderly chronic stroke patients.	Exoskeleton	Lokomat FreeD (EG) vs. conventional therapy (CG). RAGT: 60 min/session, 5 times/week for 8 weeks. Measures: FIM, Tinetti, 10MWT, HRS-D, GAS, SUS.	Within group: in EG improvement in 10MWT (d^#^ = 2.30).
Park C. et al., 2021 [24]	Comparison of humanoid robot-assisted gait training targeting multiple joints with conventional therapy.	Preliminary RCT	20 acute stroke patients.	Exoskeleton	Walkbot-based ankle–knee–hip Interlimb Coordinated robotic Training (EG) vs. conventional therapy (CG). RAGT: 30 min/session, 7 times/week for 2 weeks. Measures: gait coordination, muscle activation, FMA-LE synergy scale, MAS.	Within group: in EG improvement in active force data in the paretic limb (η2 Hip* = 0.64, η2 Knee* = 0.64, η2 Ankle* = 0.67), Peak passive stiffness (d Hip* = 0.95, d Knee* = 0.87, d Ankle* = 0.68), MAS score (d Hip Extensor = 0.35, d Knee Extensor = 0.11, d Ankle Plantar-flexor = 0.45).
Longatelli V. et al., 2021 [35]	Investigate the effects of robotic exoskeleton gait training on neuromuscular coordination and muscle activation in stroke rehabilitation.	A single-blinded pilot study	29 sub-acute stroke patients.	Exoskeleton	EKSO GTA (EG) vs. conventional therapy (CG). RAGT: 3 times/week for 12 sessions. Measures: BI, MI, 10-MWT, 6MWT, FAC, and TCT combined into a Capacity Score Gait Metric (GM) and EMG agonist-antagonist muscle coherence	Within group: in EG and CG improvement in Capacity Score (BI + MI + 10MWT + 6MWT + FAC + TCT) (d EG^#^ = 1.73, d CG^#^ = 0.83). Between group: In EG improved semitendinosus activation in both paretic and non-paretic side (d paretic = 3.00, d non-paretic = 2.41), Capacity score (d^#^ = 0.75) compared to the CG.
Park C. et al., 2020 [23]	Evaluate the effects of Walkbot-assisted robotic training on ambulation, cardiopulmonary function, depression, and fall confidence in acute hemiplegia.	RCT	14 acute stroke patients.	Exoskeleton	Walkbot-based locomotor training (EG) vs. conventional therapy (CG). RAGT: 60 min/session, 7 times/week for 2 weeks. Measures: BBS, FAC, heart rate, BRPE, BDI-II, and ABC scale	Between group: in EG improvement in FAC compared to the CG (d = 1.30).
Ogino T. et al., 2020 [37]	Investigate the effectiveness of gait training using GEAR compared to treadmill training for chronic stroke patients.	RCT	21 chronic stroke patients	Exoskeleton	Gait Exercise Assist Robot (GEAR) (EG) vs. treadmill training (CG). RAGT: 5 times/week for 4 weeks. Measures: FIM, FAC, 10-MWT, 6MWT, SF-8, GRC.	Within group: in EG improvement in gait speed at T1 (d = 0.40) and 1-mo follow-up (d = 0.37); in stride length at 1-mo follow-up (d = 0.33) and 3-mo follow-up (d = 0.36); in GCR scales at T1, 1-mo follow-up, and 3-mo follow-up (d GRC = N.C. in all conditions). In CG GRC scale increase at 1-mo follow-up (d = N.C.). Between group: in EG improvement in 6MWT (d = 1.04) at T1 compared to the CG.
Ando D. et al., 2020 [26]	To evaluate encephalic white matter microstructural changes associated with gait training using the HAL in patients initiated within 1 week of stroke onset.	Observational study	27 acute stroke patients	Exoskeleton	HAL training (FL-05) (EG) vs. conventional therapy (CG). RAGT: 3 times/week for 3 weeks. Measures: FMA, FAC, FIM, MMSE, FA, mean diffusivity, radial diffusivity and axial diffusivity images.	Within group: in EG and CG increase in FMA (d CG^#^ = 1.10, d EG^#^ = 0.94), FAC (d CG^#^ = 1.62, d EG^#^ = 2.03). No difference between group.
Rojek A. et al., 2020 [38]	Evaluate the effects of EKSO GT training on balance, load distribution, and functional status of patients after ischemic stroke.	RCT	44 chronic stroke patients	Exoskeleton	EKSO GT (EG) vs. conventional therapy (CG). RAGT: 45 min/session, 5times/week for 4 weeks. Measures: instrumental balance and load distribution values with closed and open eyes, RMI, BI, walking time and number of steps monitored with the EKSO GT Exoskeleton.	Within group: In EG increase in walking time and number of steps (d = N.C.).
Yokota C. et al., 2019 [27]	Evaluate the effects of gait training, initiated within 1 week of acute stroke onset, by using HAL.	Pilot study	37 acute stroke patients	Exoskeleton	HAL training (FL-05) (EG) vs. conventional therapy (CG). RAGT: 20 min/session of robotic training, 1–3 sessions per day, 5 or 6 times a week for at least 1 week, but up to 6 weeks, according to the patients’ achievements. Measures: FMA, FIM, FAC	Between group: improvement in FAC at 2nd (d° = 3.35) and 3rd evaluation (d° = 4.44) in more severe patients, no difference in FIM, compared to the CG.
Calabrò R.S. et al., 2018 [39]	Investigate the impact on gait training by using EKSO on gait performance and recovery of specific brain plasticity mechanisms of chronic stroke patients	RCT	40 chronic stroke patients	Exoskeleton	EKSO gait training (EG) vs. over ground gait training (CG) RAGT: 45-min/session, 5 times/week for 8 weeks. Measures: 10MWT, RMI, sEMG from lower limbs, FPEC, SMI.	Between group: in EG improvement in the 10MWT (d* = 0.90), hip and knee muscle activation (d* = 0.80) compared to the CG.
Bergmann J. et al., 2018 [44]	Assess the effects of RAGT on pusher behavior compared to non-robotic physiotherapy.	RCT	30 sub-acute stroke patients.	Exoskeleton	Lokomat gait training (EG) vs. non-robotic physiotherapy (CG). RAGT: 60 min/session, 5times/week for 2 weeks. Measures: SCP, BLS, POMA-B, FAC, SVV.	Between group: no difference in FAC.
Watanabe H. et al., 2017 [36]	Assess the effects of HAL gait training on gait performance in recovery-phase stroke patients.	RCT	22 sub-acute stroke patients	Exoskeleton	HAL single-leg version training (EG) vs. conventional therapy (CG). RAGT: 20min/session, 3 times/week for 4 weeks. Measures: FAC, 10-MWT, 6MWT, SPPB, FMA-LE, Isometric Muscle Strength (hip flexion, hip extension, knee flexion, knee extension).	Between group: In EG improvement in FAC after 12 sessions (d = 0.38), and at 8- and 12-weeks post intervention compared to the CG (d 8-weeks = 0.73, d 12-weeks = 0.55). No difference in 6MWT, FM-LE, maximal speed, stride and cadence.
Yang H. E. et al., 2017 [45]	Investigate the imaging and motor changes in post-stroke injured brains after RAGT	prospective open-label study	10 sub-acute stroke patients	Exoskeleton	Walkbot-based training (EG) at different time points. RAGT: 45min/session, 3times/week for 20 sessions. Measures: FM-LE, MI, FAC, TCT, DTI data and FA.	In EG improvement in FMLE, MI, TCT after 20 section of treatment and1-month follow-up (d = N.C.).
Taveggia G. et al., 2016 [32]	Evaluate the effectiveness of a robot training compared with a usual gait training in post-stroke hemiparesis.	RCT	28 sub-acute stroke patients	Exoskeleton	Lokomat training (EG) vs. conventional therapy (CG). RAGT: 25 treatment sessions, 5times/week for 5 weeks. Measures: 6MWT, 10-MWT, FIM, SF-36, TIN-B.	Within group: in EG improvement in 10MWT at T1 (d = 0.76) and follow-up (d = 0.80). In CG increase in 6MWT at the follow-up (d = 0.76).
Watanabe H. et al., 2014 [33]	Compare the efficacy of gait training using a single-leg version of the Hybrid Assistive Limb (HAL) on the paretic side with conventional gait training in subacute stroke patients.	RCT	22 sub-acute stroke patients	Exoskeleton	HAL single-leg version (EG) vs. conventional therapy (CG). RAGT: 20 min/session, 3 times/week for 4 weeks. Measures: FAC, maximum walking speed, 6MWT, SPPB, FM-LE, and isometric muscle strength (hip flexion and extension, knee flexion and extension).	Within group: in EG increase in FAC (d = 6.25), walking velocity (d = 2.09), 6-MWT (d = 0.38), and FM-LE (d = 2.17). In CG improvement in FAC (d = 3.00). Between group: In EG improvement in the FAC compared to the CG (d = 0.36).
Peurala S. H. et al., 2009 [28]	Analyze the effects of robotic gait therapy in acute stroke patients.	RCT	56 acute stroke patients	End-Effector	Body-weight-supported exercise on the Gait Trainer (EG) vs. walking exercise over ground (WALK) vs. conventional treatment (CG). RAGT: 20 min/session, 5times/week for 3 weeks. Measures: FAC, 10MWT, 6MWT, MMAS, RMA, RMI.	Between groups: in EG and WALK group improvement in FAC and RMI scores at T1 (WALK: d FAC^#^ = 1.35, d RMI score = 1.68; EG: d FAC^#^ = 1.00, d RMI score = 1.64) and at 6-month follow-up (WALK: d FAC^#^ = 1.25, d RMI score = 1.90; EG: d FAC^#^ = 0.65, d RMI score = 1.69) compared to the CG. Between groups: in EG improvement in 10MWT (d = 0.49) and 6MWT (d = 1.01) at T1 compared to WALK.
Dias D. et al., 2007 [40]	Compare the efficacy of gait trainer with conventional treatment on gait management after stroke.	RCT	40 chronic stroke patients.	End-Effector	Gait Trainer (EG) vs. conventional treatment (CG). RAGT: 20 min/session, 5 times/week for 5 weeks. Measures: MI, TMS, mASS, BBS, RMI, F-MSS, FAC, BI, 2meters walking test and gait cycle parameters, 6MWT, step test.	Within group: in both group improvements at T1 in MI (d EG = 0.58, d CG = 0.60), Touluse motor scale (d EG = 0.98, d CG = 0.95), RMI (d EG = 0.47, d CG = 0.69. In EG improvement in F-MSS (d = 0.55), 6MWT (d = 0.72) and 10MWT (d 10 M Step cadence = 0.49, d 10 M Step length = 0.61), at T1. In EG Improvement at follow-up in MI (d = 0.58), TMS (d = 0.62). In CG Improvement at follow-up in 10MWT (d 10M Step cadence = 0.64, d 10M Step length = 0.63), step test (d = 0.59).

Notes: d = Cohen’s Effect Size; η^2^ = Eta squared; N.C. = Not Calculable; # = Cohen’s d calculated based on median values; * = Cohen’s d from the paper results; ° = Cohen’s d from the predicted values.

Exoskeletons reported were: Walkbot, FREE walk, Hybrid Assistive Limb (HAL), Honda Walking Assist (HWA), Lokomat, EKSO-GT, Gait Exercise Assist Robot (GEAR), WellWalk, UAN.GO–OPTIGO platform. The end-effector devices used were: Movendo Hunova robotic platform, G-EO System, and Gait trainer (Table 2).

All included articles address robotic devices designed for repeatable, targeted training—essential for neuromotor learning and promoting near-physiological walking.

End-effector devices interact with the distal part of a limb (e.g., foot) to generate movement patterns that indirectly affect the whole limb. In this scoping review, the Gait Trainer (as in [28,40]) appears as the first end-effector implementation involving motorized moving treads. Subsequent innovations introduced distal fixation, as in the G-EO system (as in [31]), which incorporates body weight support and two footplates to control step length and height.

On the other hand, exoskeleton devices align with the lower limb joints and use actuators to guide hip, knee, and ankle movements, generating a preprogrammed walking cycle [13,47,48,49]. They assist walking, posture, and balance by providing controlled alignment of joints. However, although they are suitable for a variety of rehabilitation settings, they require skilled supervision and are expensive due to their complexity. These devices can be passive, relying on kinematic forces for movement, or active, using motorized actuators that target one (e.g., the HWA hip-focused as in [43]) or multiple joints (e.g., the EKSO-GT hip and knee-focused as in [35,38,39,50]).

Exoskeleton-based gait training can be performed with stationary or wearable devices. Stationary options offer repetitive, controlled training in a confined space but require specialized facilities. Both Lokomat (as in [14,32,44]), GEAR [37], and WellWalk [29] use a treadmill equipped with body weight support, each targeting different joints, while Walkbot (as in [23,24,34,41,45]) employs an ankle–knee–hip exoskeleton as a humanoid limb-coordinated robotic system. Wearable exoskeletons, on the other hand, allow over-ground walking and overcome structural limitations, but demand the patient to have adequate trunk control and require greater therapist support. The biped HAL [25] cyborg-type exoskeleton supports gait training via both voluntary and autonomous modes by detecting motor unit potentials through skin sensors and combines signal analyses of joint angles and floor reaction force to estimate the walking cycle. A single-leg version of HAL [33,36] put on the paretic side could be used, combining both mode and training exclusively on the weakened limb.

### 3.3. Motor Outcomes

#### 3.3.1. Spatiotemporal Gait Parameters

In studies reviewed, spatiotemporal gait parameters such as walking speed, stride length, and cadence, alone or extracted from 6-Minutes Walking Test (6MWT), and 10-Meters Walking Test (10MWT), were analyzed.

In [14,31,32,33,35,40,42] parameters related to endurance or walking speed were statistically improved in the experimental group (EG) compared with the baseline, however, few studies reported improvement when compared with the control group (CG) [28,35,39]. In detail, in the context of endurance (6MWT), Lee et al. [42] observed a substantial improvement in ambulatory performance, with patients showing a median increase in walking distance from 10.0 meters at baseline to 33.0 meters post-treatment. This corresponds to a 230% improvement and reflects a large effect size (Cohen’s d = 0.99), suggesting a strong clinical impact. Aprile et al. [31] similarly reported an increase in 6MWT performance, from 72.0 meters to 119.0 meters, representing a 65.3% improvement and a moderate effect size (Cohen’s d = 0.52). Supporting these results, Dias et al. [40] found an effect size of 0.72 for changes in 6MWT performance following RAGT, further reinforcing the intervention’s capacity to enhance walking endurance in neurological populations. Additionally, when comparing intervention and control groups, Ogino et al. [37] and Peurala et al. [28] found large between-group effects in favor of RAGT, with Cohen’s d values of 1.04 and 1.01, respectively.

Improvements were also evident in walking speed, as assessed by the 10-Meter Walking Test (10MWT). Manuli et al. [14] reported a significant reduction in the time needed to cover 10 meters, with median walking time decreasing from 20.70 seconds at baseline to 14.20 seconds after the intervention. This represents a 31.4% improvement and a very large effect size (Cohen’s d = 2.30), indicating meaningful gains in gait speed. Likewise, Taveggia et al. [32] demonstrated an increase in average walking velocity from 0.27 m/s to 0.56 m/s, corresponding to a 107.4% improvement and a large effect size (Cohen’s d = 0.76), pointing to substantial gains in functional ambulation.

On the other hand, using exoskeleton devices in chronic stroke patients and end-effectors in acute and sub-acute stroke patients [28,37,39] determined a statistically significant improvement in spatiotemporal gait parameters compared to CG.

Among these studies, only Calabrò et al. [39] reported effect sizes, demonstrating large effects for 10MWT (Cohen’s d = 0.90) favoring RAGT. In addition, Longatelli et al. [35] found a medium-to-large effect in favor of the RAGT in the Capacity Score (Cohen’s d = 0.75), a comprehensive index combining six clinical scales to assess motor capabilities and functional abilities, including the Barthel Index (BI), Motricity Index (MI), 10MWT, 6MWT, FAC, and Trunk Control Test (TCT).

This effect seems to persist over time as demonstrated in chronic patients who used an exoskeletal dispositive (GEAR) [37] that showed a significant increase in stride speed at the end of training (Cohen’s d = 0.38), which was sustained during the 1-month follow-up (Cohen’s d = 0.33), with further stride length improvements at 3-month follow-up (Cohen’s d = 0.36).

Interestingly, one study [43] reported that robotic gait assistance (HWA) can have immediate small effects on gait speed (Cohen’s d = 0.28) and stride length (paretic side, Cohen’s d = 0.22), supporting its potential to enhance walking efficiency in elderly stroke patients.

RAGT systems incorporating dynamic weight support mechanisms (as the Walkbot device used in [41]) have been shown to promote normalization of plantar loading asymmetries, thereby serving as a surrogate marker for enhanced weight-bearing symmetry.

Spatiotemporal gait parameters were primarily assessed through clinical evaluation, with limited use of instrumental methods such as the GAITRite^®^ system (as in [43]) and accelerometer-based systems (as in [39]).

Longatelli et al. [35] compared a powered robotic device (EKSO) with conventional care alone in subacute post-stroke patients, showing improved muscle activation. A large effect was found in the Semitendinosus muscle both for the paretic and non-paretic side (Cohen’s d 3.00 and 2.41). Similarly, Calabrò et al. [39] demonstrated that EKSO enhanced muscle recruitment with a large effect on muscle activation, particularly in the paretic Biceps Femoris and the non-paretic Rectus Femoris muscles during the stance phase in chronic stroke patients (Cohen’s d = 0.80).

#### 3.3.2. Balance and Fall Risk

In this scoping review, 14 research papers addressed the role of robotic devices in the treatment of trunk control and balance disorders following the cerebrovascular event. Data from studies are summarized in Table 3.

Two studies used platforms specifically designed for balance training, in one case the device was used in addition to an end-effector (G-EO system + Movendo Hunova^®^ robotic platform) [31] to create a training focused on the recovery of trunk/balance/walk control and a group focused only on walking; the other involved the use of a platform specifically designed for balance rehabilitation (Hunova^®^ robotic platform) [30].

When used alone, the balance platform improved all outcomes in the EG, showing greater gains than the CG, which had small and less significant improvements. In particular, trunk stability and balance under dynamic conditions improved.

Combining the balance platform with the G-EO robotic end-effector device yielded similar benefits to RAGT alone but with additional significant reductions in trunk oscillations and displacement in both open-eye and closed-eye conditions. Thus, the combination improved multidirectional trunk control and postural responses to static and dynamic balance challenges.

In Kim et al. [34], 15–18 treatment sessions of Walkbot training in sub-acute and chronic phases determined an enhanced trunk balance and coordination compared to conventional physiotherapy, but no significant differences between balance outcomes. Likewise, Rha et al. [41] showed that as few as 12 Walkbot sessions during the chronic stage were sufficient to improve trunk symmetry and reduce lateral angular asymmetry.

The TUG test is an established tool for assessing functional mobility and is an indirect measure of fall risk. In the study by Castelli et al. [30], which utilized the Hunova^®^ robotic platform, a large within-group effect was observed in the EG (Co-hen’s d = 0.94), and a medium within-group improvement was found in the CG (Co-hen’s d = 0.40). Additionally, a large between-group effect size (Cohen’s d = 0.94) was reported in favor of RAGT, confirming previous findings by Ogino et al. [37], who found a larger effect size (Cohen’s d = 1.90). Similarly, in the RCT by Yi-Heng Lee et al. [42], the EG, which used the FREE Walk exoskeleton, showed a 28% reduction in TUG time compared to baseline, with a medium effect size (Cohen’s d = 0.39), while the control group improved by only 2%, with a negligible effect (Co-hen’s d = 0.06). However, no statistically significant difference between groups was found at the end of the intervention, likely due to the short training duration (three sessions per week for four weeks).

With reduced balance and mobility, elderly stroke patients are more susceptible to fear of falling. In Park et al. [23] the analysis of the Activities-Specific Balance Confidence (ABC) scale demonstrated that using the Walkbot exoskeleton led to increased confidence in balance and improved performance in various ambulatory activities without falling, compared to conventional treatment (reported Eta squared, η2 = 0.31).

Alterations in postural control and balance in the elderly post-stroke patient may also include pusher behavior or latero-pulsion syndrome [51]. One study [44] showed that 2weeks of intensive RAGT with Lokomat device reduced pusher behavior in 40% of sub-acute patients, with lasting effects at follow-up, suggesting that upright body positioning and somatosensory stimulation help recalibrate the internal reference of verticality.

#### 3.3.3. Sensorimotor Impairment, Spasticity and Synergistic Patterns

The Fugl–Meyer Assessment (FMA) is commonly used as the primary outcome of clinical studies to assess sensorimotor impairment in patients with hemiparetic stroke. In Kim et al. [34] study, training by Walkbot device determined a relatively greater increase in FMA sensorimotor retrieval function in EG than the CG (Cohen’d not calculable). In Yang et al. [45] the Walkbot device determined an improvement also at 1-month follow-up. Instead, in [24,26,33,36] no differences between groups were found. The FMA-LE synergy scale (sub-score II index) assessed volitional or voluntary locomotor movement patterns, including flexor and extensor synergy. Park et al. [24] used the Walkbot device to attenuate abnormal spasticity and synergistic patterns in post-stroke patients. Given how joint stiffness inversely correlates with the ability to perform voluntary coordinated movements with synergy (coordination of multiple muscles), an inverse correlation between spasticity and extensor synergy was identified through FM-LE and MAS. Brunnstrom recovery stage (BRS) scale was used to describe motor recovery after stroke, evaluating the progression from flaccidity to near-normal coordination through movement synergies [52]. Degami et al. [25] observed in the early group a stage 5 of BRS, indicating a diminished role of co-movements and an enhanced control of separate movements, instead patients belonging to the late group (BRS stage 4) showed a control of detachment movements and an initial reduction of spasticity.

#### 3.3.4. Functional Ambulatory Category

The six-level FAC evaluates walking ability and the required level of support. In the studies reviewed, baseline FAC levels ranged from 0 to 3, indicating recovery stages (acute vs. subacute). Post-RAGT studies reporting FAC changes showed similar effects to control group (CG) in G-EO+Hunova Movendo [31] (Cohen’d within-group CG 0.51 EG 0.78) and HAL [26] (Cohen’d within-group CG 1.62 EG 2.03) use, and a statistically positive increase compared to the CG by using Walkbot (Cohen’d between-group = 1.30) [23] and HAL [27,33,36] devices (Cohen’d 3.35; 4.44; 0.55 at follow-up). RAGT with the single-leg version of HAL in the sub-acute phase, improves FAC more than conventional gait training, with benefits lasting 3 months [36]. Similarly, HAL training enhanced walking autonomy in acute stroke patients with severe initial walking disability, with benefits lasting 5 months (Cohen’d 4.44) [27]. No differences were observed in patients with pusher behavior [44] or those performing specific balance training [30].

### 3.4. Therapy Intensity

In literature, RAGT intensity varies by study type and context [53]. One widely adopted approach categorizes it by weekly rehabilitation time: high (7–5 times/week) and low (3 times/week) [54]. Increased therapy intensity, however defined, has been shown to improve the recovery of motor deficits following stroke [55,56,57].

In the analyzed works, weekly work intensity, defined as the number of sessions per week, varied from a single session [43] to 3 times/week [25,26,27,29,30,31,34,35,36,41,42,45], 5 times/week [14,28,32,36,37,38,39,40,44], or 7 times/week [23,24].

Only two studies applied very intensive training (7 times/week) using Walkbot-exoskeleton in the acute phase of stroke [23,24]. Patients achieved improvement in muscle hypertonia (Modified Ashworth Scale-MAS-score), emotional state (BDI-II), heart rate, and Berg rating of perceived exertion compared to conventional treatment.

Additionally, early intensive gait training, performing body-weight-supported exercise on the Gait Trainer, resulted in walking ability improvement, measured by 10MWT, 6MWT, and RMI scores, maintained at 6-month follow-up, compared to conventional treatment [28].

In the subacute phase, HAL-assisted gait training provided superior walking ability in terms of FAC score improvement compared to conventional treatment [33].

Chronic elderly post-stroke patients also benefit from high-intensity (5 times/week) RAGT by using exoskeleton (Lokomat-Pro FreeD in [14], GEAR in [37]) in terms of walking speed, gait balance, level of disability, QoL, and emotional status, compared to conventional therapy.

### 3.5. Non-Motor Outcomes

#### 3.5.1. Disability

Among 25 studies, various clinical assessment tools for disability were reported: Functional Independence Measure (FIM), Barthel Index (BI) and its modified version (mBI), and Rankin Scale (RS) and its modified version (mRS).

In Degami et al. [25], patients who received early gait training with a wearable exoskeleton (HAL) showed significant improvements in mRS scores compared to later interventions. In Yokota et al. [27] higher-dose intensive gait training combined with the HAL exoskeleton, enhanced motor and cognitive function, as measured by the FIM assessment. The between-group effect sizes were substantial (Cohen’s d = 2.85 for motor function and d = 3.55 for cognitive function) compared to CG. These large effects were particularly evident in acute patients with severe lower limb impairment, in whom the motor sub-score also improved at 3–5 months after the stroke event [27]. In the subacute stroke phase, using an end-effector device alone (G-EO) or combined with a balanced training system (Movendo) resulted in a comparable improvement in activity of daily living (ADL) independence between the two groups [31]. Conversely, Castelli et al. [30] found a large effect of the training with the Movendo Hunova robotic platform, alone, resulting in greater improvement in mBI scores than conventional treatment (Cohen’d between-group 1.77).

Hybrid exoskeleton systems supporting both voluntary and autonomous movements (HAL) improved motor and cognitive FIM subscale in acute stroke patients [26], even though this improvement was not prevalent with respect to CG. Similarly, Longatelli et al. [35] reported that EKSO-GT-mediated RAGT achieved significant improvement in disability scores comparable to conventional therapy. In this case, the mBI was included in a set of functional assessments called Capacity score, which balances the weight of the different assessments to obtain a single and unified functional improvement index. In Rojek et al. [38] EKSO-GT training led to improvements across all the Barthel index items investigated, while CG improvements were limited to only a few items. In Taveggia et al. [32] the Lokomat device led to progressive disability reduction in sub-acute patients, with significant improvements observed between 25 treatment sessions and a 3-month follow-up. In Manuli et al. [14] both FreeD Lokomat training with virtual reality (VR) integration and traditional training showed a similar reduction in disability, as measured by FIM, in elderly chronic patients. Furthermore, in chronic patients, the use of an end-effector device, such as the Gait trainer [40], resulted in an improvement in the Barthel mobility score at the end of treatment and 3-month follow-up. Only the study of Kim et al. [34], which used Walkbot exoskeleton in the subacute-chronic post-stroke phase, instead, found no statistically significant differences in the mBI.

#### 3.5.2. QoL and Psychological Well-Being

Only in 4 studies, the QoL was assessed. The self-reported outcome measures Short Form Health Survey (SF) SF-36, SF-8, SF-12, and EuroQol-5Dimension (EQ-5D) were used.

In Taveggia et al. [32] the use of SF-36 showed no statistically significant differences between the EG and the CG, even though the EG showed improvements in motor parameters. Two studies used shorter versions of the SF-36, (SF-12 and SF-8), to minimize administration time. Ogino et al. [37] found improvements in social functioning and role-physical at 1 month and 3 months after treadmill training, with positive changes in QoL, particularly in general health and emotional components at follow-up. Lee et al. [42] found significant improvement in both mental subdomain and total score for patients performing RAGT, with the total score being the most significant outcome compared to CG (Cohen’d 1.72).

In Castelli et al. [30] the evaluation of generic QoL was conducted by using the EQ-5D. In the study, both CG patients and patients who used the Hunova device showed improvement in their scores at the end of the study, with the EG prevalent over the CG in the intergroup analysis (Cohen’d within-group CG 1.00 EG 3.40).

#### 3.5.3. Perception of the Achievement of the Therapeutic Goal

Manuli et al. [14], by using the Goal Attainment Scaling (GAS) highlighted that intensive training with robotic devices allows a better motor outcome as well as a greater perception of having achieved the improvement. Furthermore, the subjects reported a high level of usability and a good perception regarding the achievement of the set objectives. In Park et al. [23] a participant satisfaction questionnaire (Client Satisfaction Questionnaire-8, CSQ-8) was used to assess client satisfaction with Walkbot treatment, receiving positive comments such as “good experience”, “extra time helped a great deal”, “Train one person instead of training multiple people to use the robot”.

In Kubilius et al. [46] the robotic device usability was rated as poor by most participants based on the System Usability Scale (SUS), although Tele-healthcare Satisfaction Questionnaire—Wearable Technology (TSQ-WT) scores suggested moderate to good user satisfaction across conditions.

#### 3.5.4. Microstructural White Matter Changes and Neuroplasticity

In Ando et al. [26] the use of HAL in the acute phase of stroke determined microstructural changes in white matter in comparison with the conventional physical therapy, as indicated by DTI-MRI. MRI conducted within 2 weeks of stroke onset and at 3–5 months after onset showed a lower Fractional Anisotropy (FA) of the ipsi-lesional cerebral peduncle in the CG, reflecting the pathological process of Wallerian degeneration after stroke onset, versus an increase in the contra-lesional rostrum of the corpus callosum in the HAL, indicating the restoration of interhemispheric communication which was reduced at baseline after stroke onset.

Yang et al. [45] conducted an MRI study at baseline and the end of RAGT by using a Walkbot device in 10 post-stroke patients in the early sub-acute phase. Treatment showed a significant increase in FA values in the supplementary motor area and supra-marginal gyrus of the unaffected hemisphere, and the posterior cingulate cortex of the affected hemisphere. Conversely, FA values in the internal capsule, substantia nigra, the pedunculopontine nucleus of the affected hemisphere, and the middle temporal area of the unaffected hemisphere significantly decreased.

Calabrò et al. [39] found that, in a chronic stroke stage, the use of EKSO determined a rebalance of the cortico-spinal integration of the affected and unaffected hemispheres, in parallel to cortico-spinal excitability interhemispheric remodulation, becoming the most important factors correlated with the clinical improvement.

#### 3.5.5. Cognitive, Emotional, and Miscellaneous Functions

In the retrospective study of Kim et al. [34] focused on post-stroke patients with dementia, the combination of verbal cognitive tasks and locomotor training with the Walkbot system, determined a relatively greater improvement in Mini-Mental State Examination (MMSE) cognitive function in the EG than in the CG.

In the Castelli et al. study [30], the use of a robotic platform for balance training (Hunova) in sub-acute post-stroke patients showed improvement in executive functions, information processing speed, attention, and discrimination of multiple stimuli.

Park et al. [23] found that Walkbot training changed the Beck Depression Inventory-II (BDI-II), but within a 0–13 range, indicating no or minimal depression and suggesting the absence of mood disorders in participants. However, robotic training may have enhanced motivation and engagement. Similarly, Manuli et al. [14] reported a significant mood improvement in the EG following Lokomat training.

In Aprile et al. [31] G-EO training reduced pain, despite the unspecified pain location. Park et al. [23] found improved cardiovascular endurance after Walkbot use. In the study by Kubilius et al. [46], the combination of RAGT with exoskeleton and walker support resulted in significant differences in heart rate and R–R interval during the sit-to-stand task compared to the control condition.

Castelli et al. [30] observed significant fatigue reduction using the Hunova platform. Peurala et al. [28], showed that RAGT was less fatiguing than over-ground walking, likely due to weight-relief harnesses and motorized platforms reducing effort. Moreover, the application of the UAN.GO wearable exoskeleton (as in [46]) produced inconsistent findings regarding perceived fatigue and workload, as measured by the NASA-TLX, with a modest improvement noted under comfortable walking speed conditions when walker support was employed.

#### 3.5.6. Combination of RAGT with Virtual/Augmented Reality

In Park et al. [24] the humanoid robotic system was integrated with virtual and augmented reality (VR/AR) games and scenarios to maximize patient interest, motivation, and active involvement while reducing anxiety and depression during therapy sessions. Furthermore, Kim et al. in [34] used the Walkbot system in post-stroke dementia to combine cognitive and locomotor exercises by using AR and VR.

## 4. Discussion

This scoping review aims to evaluate the use of robotic devices for gait rehabilitation in elderly post-stroke individuals, with a focus on both motor and non-motor effects. The predominant findings relate to motor improvements, such as increased gait independence, changes in gait parameters, and enhanced balance control. Additionally, notable non-motor effects were observed, particularly in QoL, cognitive function, and neuroplasticity.

The studies analyzed showed a prevalence of exoskeleton use compared to end-effector devices, with a higher percentage in the subacute phase and a lower percentage in the acute and chronic phases. Thus, it appears a prevalence of robotic devices’ applications during the stabilizing stage of the disease, when some predefined motor goals, such as trunk control and partial dependence on walking, have been achieved. Despite the evidence supporting the efficacy of end-effector devices in the rehabilitation of elderly stroke patients, the findings of this scoping review show that their use remains significantly limited compared to exoskeletons. This imbalance likely stems from both methodological preferences and a lack of targeted research in the elderly.

Although men are generally at a higher risk of stroke during most of their lifespan, the incidence in women increases significantly with age, particularly after menopause, and rises sharply beyond 85 years. When combined with women’s longer life expectancy, this leads to a higher number of stroke-related deaths among women compared to men [58]. Despite this, women remain underrepresented in rehabilitation research, as reflected by their limited inclusion in the present review. This discrepancy underscores the need for more inclusive studies that better represent the true demographics of the elderly stroke population.

The evidence emerging from the studies analyzed suggests that early initiation of RAGT in elderly post-stroke patients significantly enhances motor recovery and quality of life, with the greatest benefits observed in those with severe lower limb impairments. Although improvements in balance remain modest, early exoskeleton-based training accelerates the achievement of supervised gait and shortens rehabilitation stays—a crucial outcome for older adults at high risk of complications from prolonged immobility. A recent Cochrane review suggests that acute and early sub-acute stroke patients may profit from RAGT more than late sub-acute and chronic stroke patients [17]. Furthermore, treadmill-based exoskeleton gait training yields greater improvement when started in the acute phase than in later stages [59]. Conversely, RAGT seems to be equivalent to traditional therapy for chronic stroke patients [60,61]. Therefore, given the comorbidities and reduced cognitive reserve in the elderly stroke population, starting the RAGT at the earliest feasible stage is essential to support optimal rehabilitation outcomes.

The 2016 AHA guidelines emphasize time-intensive, repetitive task training (Class I; Level A) for post-stroke patients [62]. Though there is a common belief that elderly patients should receive less intensive therapy, literature shows that advanced age does not lessen the benefits of high-intensity rehabilitation [55,56,57]. Indeed, evidence in the reviewed articles indicates that higher intensity rehabilitation (7–5 times/week), even in older stroke patients, can result in meaningful gains in motor function, gait, and overall recovery—either in the acute, sub-acute, or chronic post-stroke phase.

Rehabilitation should aim to achieve two goals: induce functional improvements -in terms of speed, endurance, safety, etc.- and guide the patient toward the recovery of rhythmic and efficient gait control -expressed as muscle activation patterns throughout the gait cycle-. Several studies have analyzed spatiotemporal gait parameters and improvements were observed primarily in walking endurance and gait speed, with several studies reporting statistically significant within-group changes following intervention. Notably, while improvements compared to control groups were less consistently reported, a subset of studies demonstrated moderate to large between-group effect sizes in favor of RAGT with immediate and prolonged effects. Endurance, typically assessed via the 6MWT, emerged as a key domain of improvement, suggesting enhanced ambulatory capacity and cardiovascular engagement in response to robotic training. Gait speed, evaluated by 10MWT, also showed consistent gains, indicating meaningful progress in functional mobility—a critical predictor of independence in post-stroke populations.

After rehabilitation, post-stroke patients usually regain the ability to walk, often relying on compensatory strategies, resulting in inefficient, non-rhythmic, and asymmetrical gait patterns, abnormally involving the unaffected ipsilesional limb [63]. In Longatelli et al. [35] and Calabrò et al. [39] studies, motor re-learning, and more efficient symmetrical gait after RAGT were observed. Restoring a symmetrical gait pattern after a stroke critically depends on the patient’s ability to recover balanced load distribution between the limbs. However, in elderly individuals pre-existing musculoskeletal and systemic comorbidities can worsen gait asymmetries and functional deficits, hindering neuromuscular recovery and increasing the risk of abnormal mobility outcomes [64]. To address these challenges, the studies reviewed suggest that wearable exoskeletons may promote more efficient and symmetrical gait patterns in elderly post-stroke patients through powered, time-synchronized, and finely modulated assistance. In particular, RAGT systems that incorporate dynamic weight support have demonstrated efficacy in normalizing plantar loading asymmetries, which may serve as a surrogate marker for weight-bearing improvement.

Primary stroke-related deficits, including sensorimotor and cognitive impairments, can increase the risk of falls—a concern that persists even in the chronic phase, particularly among elderly individuals who, despite regaining ambulation, may struggle with unexpected environmental challenges such as slips or trips [65]. In this context, early trunk control plays a pivotal role, as it underpins postural stability, supports balance, and promotes autonomy in daily living. Its targeted rehabilitation is therefore essential, given its strong predictive value for functional recovery and ADL performance [66]. The training aimed at recovering trunk control, balance, and gait can benefit from a combination of platforms specifically designed for balance rehabilitation and end-effector-type robotic devices for the lower limbs. This combination has been shown to improve multidirectional trunk control and postural responses to external perturbations of static and dynamic balance, as demonstrated by Aprile et al. [31]. Furthermore, RAGT alone in elderly patients can improve balance and trunk coordination, presumably because the robotic system provides trunk stabilization, coordinated interlimb ankle–knee–hip joint locomotor movement guidance, and associated proprioceptive and somatosensory feedback.

Reduced balance and mobility make elderly stroke patients more susceptible to fear of falling, leading to excessive activity limitation, instability, and the possibility of recurrent falls, forming a self-reinforcing vicious cycle [67]. Exoskeleton-based RAGT, by improving postural control and providing safe and accurate kinematic and kinetic control of the lower limb segments, would appear to have a positive impact on the fear of falling, as shown by Park et al. [23].

Post-stroke elderly patients may experience pusher behavior, a postural control disorder characterized by actively pushing away from the nonparetic side while resisting passive correction and a tendency to fall toward the paralyzed side. This is linked to vertical perception dysfunction and is associated with longer rehab stays and poorer outcomes [68,69,70]. One study examined this component in detail, observing that 2 weeks of intensive RAGT results in its persistent reduction for up to 2 weeks. The authors hypothesized that forced control of the upright position by the RAGT and somatosensory stimulation during locomotion can recalibrate postural reactive control as an effective method to reduce pushing behavior [44].

Strategies of reactive balance response are affected by perturbation intensity and sensorimotor abilities of an individual [71]. Furthermore, factors such as spasticity and synergistic patterns negatively affect sensorimotor control. Although different clinical assessment scales were used across the reviewed studies, and results regarding sensorimotor recovery were mixed, the use of treadmill-based exoskeleton devices—such as the Walkbot device—was generally associated with favorable outcomes [34,45]. Moreover, it is observed that, as the capacity for coordinated voluntary movement increases, joint stiffness tends to decrease. Therefore, robotic devices could reduce spasticity, associated stiffness, and abnormal synergistic extensor gait patterns [24].

The FAC is a key measure for assessing walking ability, guiding personalized rehabilitation, and tracking progress over time. The large effect sizes observed, particularly in studies employing exoskeleton devices, underscore the clinical relevance of RAGT in enhancing gait autonomy, especially in patients with severe baseline impairment (FAC < 2), and support the conclusion that stationary RAGT is more effective for stroke patients with FAC < 2 while over-ground RAGT suits more those with FAC ≥ 2 [13].

Assessing an individual’s ability to perform and complete ADLs provides insight into their overall functional status. ADL ability predicts nursing home admission, need for home assistance, and hospitalization, which can further impair independence, especially in older adults, leading to hospital-associated disability [72]. Additionally, ADL outcome measures can help assess the effectiveness of a rehabilitation treatment program [73]. Despite the heterogeneity of assessment tools used across studies, evidence consistently demonstrates improvements in ADL following RAGT. These benefits are larger when RAGT is implemented early in the rehabilitation process, administered at higher intensities, and applied to acute patients with severe impairments. This is particularly crucial for elderly patients, as aging and chronic illnesses contribute to a decline in physical abilities, including independent ADL performance. [73].

Whereas the QoL of elderly post-stroke patients is lower than that of the general population and there is limited evidence on non-pharmacological interventions in improving it, the assessment of QoL following robotic-based rehabilitation reveals promising trends but also highlights limitations [74,75]. While improvements were noted, especially in mental and emotional domains [37,42] the absence of statistically significant results in some studies [32] raises questions about the sensitivity of these measures to detect subtle changes in quality of life after rehabilitation. Variability in findings may be attributed to differences in measurement tools, sample sizes, and intervention durations. The use of shorter forms (SF-8 and SF-12) appears advantageous for reducing participant burden, but their effectiveness in capturing long-term outcomes remains unclear. Notably, follow-up assessments demonstrated delayed benefits, particularly in emotional well-being [37], suggesting that quality of life improvements may manifest over time rather than immediately post-treatment.

Robotic technology in post-stroke rehabilitation offers promising motor recovery but poses operational challenges like installation time, device weight, and therapists’ guidance while showing improved outcomes, high usability, and strong patient satisfaction [76]. Among the studies analyzed, only three specifically addressed these aspects, reporting a greater perceived sense of improvement and moderate to good levels of user satisfaction with the use of robotic devices [14,23,46]. High satisfaction levels and willingness to continue using these technologies reinforce their potential as effective rehabilitation tools, particularly in elderly stroke patients.

Traditional stroke rehabilitation has focused on compensatory strategies to manage impairments, but increased awareness of neuroplasticity has shifted the approach [77]. Stroke recovery involves brain reorganization in both hemispheres; increased activity on the affected side is linked to better outcomes, while the unaffected hemisphere can have both supportive and inhibitory roles. The process varies based on stroke timing, lesion size, and patient age [78]. What emerges from the studies analyzed is that in acute stroke elderly patients RAGT could promote brain reorganization by facilitating the recovery of inter-hemispheric communication, preventing degeneration, and enhancing plasticity in the affected pyramidal tract [26]. A different view is observed instead in an early sub-acute phase where RAGT could facilitate plasticity in the intact supplementary motor area, but not in the injured motor-related areas [45]. In a chronic post-stroke stage, RAGT improved cortico-spinal excitability and integration on the affected side, promoted interhemispheric remodeling, and reshaped corticospinal excitability in both hemispheres, unlike conventional gait training, suggesting a top-down control of motor function recovery [39]. These effects are crucial given age-related and stroke-induced brain changes.

RAGT is a promising intervention for elderly post-stroke patients not only for motor recovery but also for the potential impact on non-motor outcomes such as cognition, depression, fatigue, pain, and cardiovascular health. Cognitive deficits are often exclusion criteria in experimental studies, however, the retrospective study by Kim et al. [34] focusing on post-stroke patients with mild to moderate cognitive impairment, showed that combining verbal cognitive tasks with locomotor training resulted in relatively greater improvements in MMSE scores. Considering the interdependence of cognitive, motor, and balance functions, in the Castelli et al. study [30], the use of a robotic platform for balance training showed improvement in executive functions, information processing speed, attention, and discrimination of multiple stimuli. These outcomes support coupling cognitive and motor training to foster neuroplasticity, especially in patients with cognitive impairment, emphasizing the role of multimodal rehabilitation strategies.

Depression affects one-third of stroke survivors, hindering recovery and worsening outcomes, with a reciprocal relationship between depression and physical function [79]. Only 2 studies addressed the emotional impact of robotic devices, indicating a possibly enhanced motivation and engagement, and mood improvement [14,23]. Thus, since depression can hinder rehabilitation, addressing it through RAGT could enhance recovery and optimize outcomes, especially in older individuals.

RAGT can improve gait and cardiopulmonary fitness [23]. In particular, it appears to enable longer walking sessions without cardiovascular strain, especially in stroke patients with moderate to severe gait impairments, according to findings from the analyzed studies [46]. Given that constant moderate-intensity exercise under cardiac monitoring is key to cardiovascular reconditioning, RAGT may offer benefits for gait and cardiopulmonary fitness in post-stroke patients—particularly in the elderly, who are often excluded from rehabilitation due to cardiovascular comorbidities [80]. Furthermore, RAGT has the potential to reduce perceived fatigue during gait training in post-stroke populations [28,30]. This effect may be particularly advantageous for elderly patients, who are often limited by cardiovascular and musculoskeletal comorbidities that exacerbate exercise intolerance. However, the heterogeneity in device performance and patient responses highlights the need for individualized assessment and careful patient selection to optimize outcomes.

Pairing RAGT with VR/AR elements can increase patient engagement, motivation, and overall enjoyment, potentially amplifying therapeutic benefits [24]. It creates an immersive, gamified environment that enhances motivation and engagement in rehabilitation. Real-time feedback helps patients set and achieve clear goals, while the playful nature of VR/AR reduces the perceived difficulty and fatigue of therapy. This increased motivation improves adherence to rehabilitation protocols, promoting frequent practice, better motor learning, and ultimately, enhanced functional recovery after stroke.

Despite the growing evidence supporting the RAGT benefits in elderly post-stroke patients, its long-term effects are under-explored. Only 8 out of 25 studies evaluated effects at 2 weeks or at 1, 2, 3, or 6 months after the end of treatment [27,28,32,36,37,40,44,45]. This highlights a methodological gap, limiting the strength of current evidence on the durability of therapeutic benefits.

**Figure 3:**
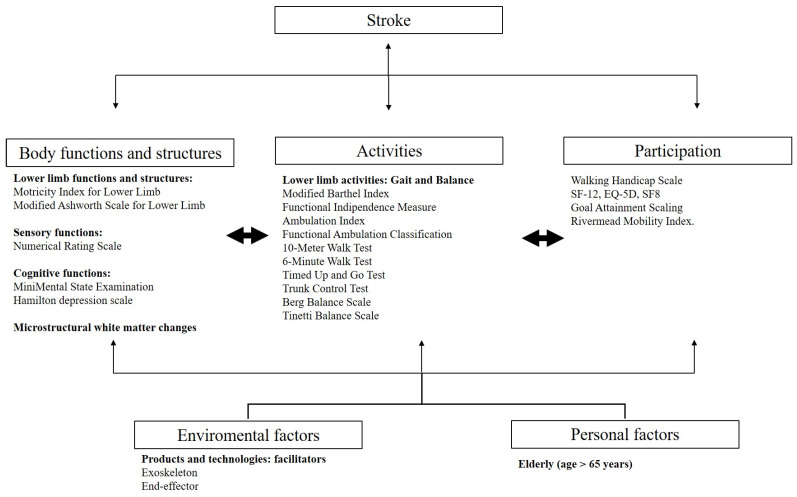
ICF Conceptual framework illustrating the interplay between stroke and its impact on body functions/structures, activities, and participation. The figure also highlights relevant assessment tools along with environmental and personal factors—such as the use of exoskeleton or end-effector technologies and older age (>65 years)—that can influence rehabilitation outcomes.

Finally, the outcomes reported in the studies included in this review highlight that RAGT impacts multiple International Classification of Functioning, Disability, and Health (ICF) domains, improving motor, sensory, and cognitive functions, as well as walking, balance, and daily living [81,82]. These improvements contribute to greater autonomy and QoL—as summarized in Figure 3.

## 5. Future Directions for Research and Suggestions for Clinical Practice

From our data synthesis, we have identified several research directions concerning the use of RAGT in elderly post-stroke populations. These are summarized as open questions to guide future investigations:

1. Which clinical and demographic characteristics—such as age strata, comorbidities, cognitive status, or severity of motor deficits—are most predictive of a positive response to RAGT in elderly stroke survivors?

Understanding individual profiles that influence responsiveness to RAGT could enable the development of personalized treatment pathways and optimize clinical outcomes.

2. To what extent is RAGT cost-effective compared to conventional physiotherapy in elderly post-stroke patients, particularly in relation to long-term functional independence, reduction in institutionalization, and quality of life?

A comprehensive cost-benefit analysis is essential to support widespread clinical adoption and ensure the sustainability of RAGT programs in geriatric rehabilitation.

3. Can the integration of emerging technologies such as virtual/augmented reality and artificial intelligence enhance the efficacy, adherence, and cognitive stimulation associated with RAGT in older adults?

Exploring the synergistic potential of multimodal systems may unlock new opportunities for engaging, adaptable, and more effective rehabilitation approaches in complex elderly populations.

In clinical practice, older stroke survivors often present with a complex array of comorbidities—such as lower limb fractures requiring prosthetic implantation, severe cardiovascular decompensation, osteoporosis, and cognitive decline—that reflect the multifaceted nature of aging-related disability. Paradoxically, these same conditions frequently constitute exclusion criteria in studies and protocols involving RAGT with exoskeletons. Consequently, some of the patients who could benefit most from technologically advanced rehabilitation approaches are systematically underrepresented or excluded. This gap highlights the critical need to identify RAGT protocols that are best suited to the clinical realities of geriatric stroke populations.

An additional methodological recommendation concerns the reporting of effect sizes and raw data. During this review, calculating effect size proved challenging in several studies due to the absence of sufficient statistical information, such as means, standard deviations, or confidence intervals. In many cases, effect sizes—especially for within-group or between-group comparisons—were either not reported or not computable from the data provided. To enhance transparency, and reproducibility, and facilitate future evidence synthesis (e.g., meta-analyses), we strongly encourage authors to report standardized effect sizes (e.g., Cohen’s d, Hedges’ g) and to make raw outcome data—or at least group-level descriptive statistics—readily available. This would enable more accurate comparisons across studies and support the development of evidence-based clinical guidelines.

## 6. Conclusions

RAGT is a valuable rehabilitation tool for elderly stroke patients, aiding in gait recovery and functional independence.

### 6.1. Age Considerations

Age-related changes, such as reduced muscle mass, impaired balance, and cognitive decline, can affect rehabilitation. While younger stroke survivors may recover faster, elderly individuals still benefit from RAGT when interventions are adjusted to their functional levels. Due to multimorbidity in older patients, RAGT should be part of a multidisciplinary strategy addressing both motor and non-motor impairments.

### 6.2. Phase of Stroke Recovery

Timing of RAGT initiation is the key to optimizing outcomes. Early intervention, especially in the acute and subacute phases, promotes neuroplasticity and functional recovery. However, in the elderly, factors like medical stability, cognitive function, and mobility limitations must be considered when deciding the best time for RAGT. While subacute patients may benefit from more intensive therapy, chronic-phase patients can still see improvements with tailored training. Overall, early initiation of RAGT in elderly post-stroke patients significantly enhances motor recovery and quality of life, with the greatest benefits observed in those with severe lower limb impairments. Early RAGT accelerates the achievement of supervised gait and shortens rehabilitation stays—crucial outcomes for older adults at high risk of complications from prolonged immobility.

### 6.3. Training Intensity and Clinical Implications

RAGT typically focuses on high-intensity, repetitive training to enhance motor learning. However, elderly stroke patients may have limited tolerance for long or intense sessions. Adjusting training duration, frequency, and robotic assistance levels is necessary to ensure adherence while minimizing fatigue. Individualized progression plans and periodic assessments will help optimize RAGT’s benefits in elderly patients.

## 7. Limitations

Despite promising findings, several limitations need to be addressed. The variability in robotic devices, training protocols, and outcome measures across studies makes it challenging to compare results directly. Many studies involved heterogeneous and relatively small sample sizes, which limits the generalizability of the findings. Numerous investigations focus predominantly on relatively younger elderly populations (i.e., patients in their 60s and early 70s), often excluding individuals with multiple comorbidities or significant cognitive impairments, which limits the generalizability of the findings to the broader, frailer elderly stroke population.

The limited inclusion of women in this review underscores their ongoing underrepresentation in rehabilitation research and the need for studies better reflecting elderly stroke global demographics.

Furthermore, there remains uncertainty regarding the long-term duration of the benefits of RAGT and its impact on the quality of life and psychological well-being of elderly post-stroke individuals. This variability in patient profiles and intervention protocols complicates direct comparisons and may have contributed to inconsistencies across study outcomes. Therefore, conclusions should be interpreted with caution, and future research would benefit from more homogeneous study designs and clearly stratified patient populations.

Finally, this scoping review does not include a critical methodological or statistical appraisal of individual study outcomes, as such analyses were beyond its primary scope. The review aims to provide a broad synthesis of RAGT applications, as well as highlight the limited development of the RAGT field in elderly post-stroke populations, encompassing both motor and non-motor domains.

By addressing these gaps, robotic-assisted rehabilitation has the potential to revolutionize elderly stroke rehabilitation, improving not only physical recovery but also cognitive and psychological well-being, ultimately enhancing patients’ QoL and functional independence.

## Figures and Tables

**Table 2:** Technical features of the RAGT devices used. Manufacturer details, type of device (exoskeleton vs. end-effector), actuated joints, EMG control (yes/no), usage environment (stationary vs. over ground), presence of a weight support system (yes/no), and references from the present review that employed each device were described.

Device Name	Company	City/Country	Type	Actuated Joint	EMG Controlled	Stationary/ Overground	Weight Support System	References
Walkbot	P&S Mechanics	Seoul, Republic of Korea	Exoskeleton	Hip/Knee/Ankle	No	Stationary	Yes	[23,24,34,41,45]
FREE walk exoskeleton device	Free Bionics Taiwan Inc.	Taiwan	Exoskeleton	Hip/Knee	No	Overground	No	[42]
Hybrid Assistive Limb lower limb type for Well-Being Lower Limb Type, biped non medical model FL-05, biped non medical model HAL single-leg version	Cyberdyne, Inc.	Ibaraki, Japan	Exoskeleton	Hip/Knee	Yes	Overground	No	[25,26,27,33,36]
Honda Walking Assist	Honda R&D Co. Ltd.	Tokyo, Japan	Exoskeleton	Hip	No	Overground	No	[43]
Lokomat Lokomat FreeD	Hocoma	Zurich, Switzerland	Exoskeleton	Hip/knee	No	Stationary	Yes	[14,32,44]
EKSO GT	EKSO Bionics Holdings, Inc.	San Rafael, CA	Exoskeleton	Hip/knee	No	Overground	No	[35,38,39]
Gait Exercise Assist Robot (GEAR) WellWalk WW-2000	Fujita Health University and Toyota Motor Corporation	Japan	Exoskeleton	Knee	No	Stationary	Yes	[29,37]
UAN.GO–OPTIGO platform	U&O s.r.l.	Fiorenzuola d’Arda, Italy	Exoskeleton	Hip/knee	No	Overground	No	[46]
G-EO System	Reha Technology AG	Switzerland	End-effector	N.A.	No	Stationary	Yes	[31]
Movendo Hunova robotic platform	Movendo Technology S.r.l.	Italy	End-effector	N.A.	No	Stationary	No	[31]
Gait trainer	Reha-Stim, Berlin,	Germany	End-effector	N.A.	No	Stationary	Yes	[28,40]

All included articles address robotic devices designed for repeatable, targeted training—essential for neuromotor learning and promoting near-physiological walking.

**Table 3:** Summary of functional results focused on balance control and fall risk, quality of life and level of disability, cognitive and emotional functions, and additional findings. Each row compares results within the experimental group (EG) and control group (CG), highlighting improvements in measures used.

Authors	Device	Balance Control and Fall Risk	Quality of Life and Level of Disability	Cognitive and Emotional Functions	Other
Rha Y.H. et al., 2025 [41]	Exoskeleton	Within group: in EG improvement in trunk symmetry variables (d STA = 1.00, d TLBA = 0.90, d TA = 1.00). Between group: in EG time-resolved improvement in shoulder tilt angle (d = 0.49), trunk lateral bed angle (d = 0.61), trunk angle (d = 0.49).	Not reported	Not reported	Not reported
Kubilius R. et al., 2024 [46]	Exoskeleton	Not reported	Not reported	Not reported	Between groups: Difference in heart rate and R–R interval in sit-to-stand w/o exoskeleton condition vs. UAN.GO (d HR = 0.34, d R-R interval = 0.49) and UAN.GO-OPTIGO (d HR = 0.14, d R-R interval = 0.48); improvement in NASA-TLX in comfortable speed walking condition with UAN.GO-OPTIGO vs. UAN.GO (d = 0.87). No difference in TSQ-WT questionnaire scores; difference in SUS score in UAN.GO vs. UAN.GO-OPTIGO use (d^#^ = 1.12).
Kim Y. et al., 2023 [34]	Exoskeleton	Within group: Increase in TIS trunk balance and coordination in both EG and CG. No significant differences between BBS outcomes. Between group: In EG increase in TIS trunk balance and coordination compared to the CG (d = N:C: for all variables).	Between group: no differences in mBI.	Between group: In EG improvement in the MMSE cognitive function (d = N.C.).	Not reported
Lee Y.H. et al., 2023 [42]	Exoskeleton	Within group: in EG improvement in TUG (d^#^ = 0.39).	Between group: in EG increase in mental subdomain (d^#^ = 0.27) and total scores (d^#^ = 1.72) of SF-12 compared to the CG.	Not reported	Not reported
Degami A. et al., 2023 [25]	Exoskeleton	Not reported	Between group: in EG improvement in mRS in the early group than that in the late group (d^#^ = 0.45). No difference in FIM	Not reported	Not reported
Castelli L. et al., 2023 [30]	End-Effector	Within group: in EG improvement in TUG (d^#^ = 0.94), BBS (d^#^ = 1.62), SPPB (d^#^ = 2.34). In CG improvement in TUG (d^#^ = 0.40), SPPB (d^#^ = 0.71). Between group: in EG improvement in TUG (d^#^ = 0.94), BBS (d^#^ = 0.45), SPPB (d^#^ = 0.85) compared to the CG.	Within group: in EG and CG improvement in mBI (d EG^#^ = 8.77, d CG^#^ = 4.72), EQ-5D (d EG^#^ = 3.40, d CG^#^ = 1.00). Between group: in EG improvement in mBI (d^#^ = 1.77) compared to the CG.	Within group: in EG improvement in FAB (d^#^ = 3.88), SDMT (d^#^ = 1.04), DCT (d^#^ = 1.56), SCWT (d^#^ = 0.49). In CG improvement in FAB (d^#^ = 1.3), DCT (d^#^ = 0.33), SCWT (d^#^ = 0.61). Between group: in EG improvement in FAB (d^#^ = 1.18), SDMT (d^#^ = 0.94), DCT (d^#^ = 0.58) and SCWT (d^#^ = 0.38) compared to the CG.	Within group: in EG and CG improvement in MFIS (d EG^#^ = 2.92, d CG^#^ = 1.15), FSMC (d EG^#^ = 0.95, d CG^#^ = 0.20). Between group: in EG improvement in MFIS compared to the CG (d^#^ = 2.84).
Aprile I. et al., 2022 [31]	End-Effector	Within group: in EG improvement in TUG (d^#^ = 0.43), TIN-B (d^#^ = 0.15), BBS (d^#^ = 0.36) and TCT (d^#^ = 0.38). In CG improvement in TIN-B (d^#^ = 0.67), BBS (d^#^ = 0.64).	Within group: in EG improvement in mBI (d^#^ = 0.91). In CG improvement in mBI (d^#^ = 0.78).	Not reported	In CG improvement in pain NRS (d^#^ = 0.59).
Manuli A. et al., 2021 [14]	Exoskeleton	Within group: in EG and CG improvement in Tinetti test (d EG^#^ = 2.79, d CG^#^ = 2.02). Between group: in EG improvement in Tinetti test compared to the CG (d^#^ = 1.49).	Within group: in EG and CG in FIM (d EG^#^ = 1.85, d CG^#^ = 0.67), GAS (d^#^ = 3.94, d CG^#^ = 11.42). Between group: in EG improvement in FIM compared to the CG (d^#^ = 0.62).	Within group: in EG improvement in HRS-D (d^#^ = 1.11). Between group: in EG improvement in HRS-D compared to the CG (d^#^ = 0.76).	Not reported
Longatelli V. et al., 2021 [35]	Exoskeleton	Not reported	Within group: in EG and CG improvement in Capacity Score (BI + MI + 10MWT + 6MWT + FAC + TCT) (d EG^#^ = 1.73, d CG^#^ = 0.83).	Not reported	Not reported
Park C. et al., 2020 [23]	Exoskeleton	Between group: in EG improvement in ABC scale compared to the CG (d = 0.90). No difference in BBS.	Not reported	Between group: in EG improvement in BDI-II compared to the CG (d = 0.79).	Between group: in EG improvement in heart rate (d = 1.25), Borg rating of perceived exertion (BRPE, d = 1.06) compared to the CG.
Ogino T. et al., 2020 [37]	Exoskeleton	Between group: in EG improvement in TUG at T1 compared to the CG (d = 1.90).	Within group: in EG improvement in SF8 at 1-mo (d = 0.78) and 3-mo (d = 0.96) follow-up. In CG improvement in SF8 at 1-mo follow-up (d = 0.20).	Not reported	Not reported
Ando D. et al., 2020 [26]	Exoskeleton	Not reported	Within group: in EG and CG improvement in FIM (d EG^#^ = 3.34, d CG^#^ = 4.50).	Not reported	Within group: in EG increase in FA of corpus callosum (d = 2.00). In CG decrease in FA of the ipsi-lesional cerebral peduncle (d = 1.31).
Rojek A. et al., 2020 [38]	Exoskeleton	Within group: in EG improvement in COP deviation (d *x*-axis* = 0.54, d *y*-axis* = 0.51). In CG improvement in COP path length (d* = 0.70), COP average velocity (d* = 0.74), COP deviation (d *x*-axis* = 0.34, d *y*-axis* = 0.29), forefoot load (d* = 0.46), backfoot load (d* = 0.41). Between group: in EG improvement in COP deviation (d *x*-axis* = 0.20, d *y*-axis* = 0.93) compared to the CG.	Within group: in EG improvement in all items of BI (d BI Total^#^ = 0.86), Rivermead Mobility Index (d RMI Total^#^ = 0.65). In CG improvement in Rivermead Mobility Index (d RMI Total* = 0.38). Between group: in EG improvement in BI (d BI Total^#^ = 2.41), Rivermead Mobility (d RMI Total^#^ = 1.26) Index compared to the CG.	Not reported	Not reported
Yokota C. et al., 2019 [27]	Exoskeleton	Not reported	Between group: in all patients EG improvement in FIM total score at 2nd evaluation compared to CG (d° = 2.88); in severe walking disability group improvement in FIM at 2nd (d° = 4.66) and 3rd (d° = 2.85) evaluation in motor subscore, at 2nd evaluation in cognitive subscore compared to the CG (d° = 3.55).	Not reported	Not reported
Calabrò R.S. et al., 2018 [39]	Exoskeleton	Between group: in EG improvement in TUG compared to the CG (d° = 0.70).	Not reported	Not reported	Between group: in EG improvement in cortico-spinal excitability in the affected side (d° = 0.50), cortico-spinal integration in the affected side (d° = 0.50) and frontoparietal effective connectivity (d° = 0.80).
Bergmann J. et al., 2018 [44]	Exoskeleton	Within group: In EG improvement in SCP and BLS at T1 and at 2 weeks follow up (SCP: d T1^#^ = 0.73, d 2-weeks^#^ = 1.16; BLS: d T1^#^ = 0.97, d 2-weeks^#^ = 0.86), improvement in POMA-B at T1 (d^#^ = 0.1). In CG improvement in BLS (d^#^ = 1.08) at 2 weeks follow up.	Not reported	Not reported	Not reported
Yang H. E. et al., 2017 [45]	Exoskeleton	Not reported	Not reported	Not reported	In EG increase in FA values in the supplementary motor area and supramarginal gyrus of the unaffected hemisphere, and the posterior cingulate cortex of the affected hemisphere; decrease in FA values in the internal capsule, substantia nigra, the pedunculopontine nucleus of the affected hemisphere, and the middle temporal area of the unaffected hemisphere (d = N.C. for all variables).
Taveggia G. et al., 2016 [32]	Exoskeleton	Within group: in EG end CG improvement in Tinetti at T1 and follow-up (d EG = 0.75, d CG = 1.03).	Within group: in EG improvement in FIM at the end of treatment (d = 0.9) and 3mo follow up (d = 1.10). No differences in SF36.	Not reported	Not reported
Watanabe H. et al., 2014 [33]	Exoskeleton	Within group: in EG increase in TUG test score (d = 0.89), in CG improvement in TUG (d = 0.69), SPPB balance (d = 0.36).	Not reported	Not reported	Not reported
Dias D. et al., 2007 [40]	End-Effector	Within group: in both group improvement in BBS at T1 (d EG = 0.60, d CG = 0.51). In EG improvement at follow-up in BBS (d = 0.87).	Within group: in EG improvement at follow-up in Barthel mobility score (d = 0.54).	Not reported	Not reported
Peurala S. H. et al., 2009 [28]	End-Effector	Not reported	Not reported	Not reported	Between groups: The effort required to achieve the results measured by Borg scale were reduced in the EG group (d = 1.00).

Notes: d = Cohen’s Effect Size; η^2^ = Eta squared; N.C. = Not Calculable; # = Cohen’s d calculated based on median values; * = Cohen’s d from the paper results; ° = Cohen’s d from the predicted values.

## Data Availability

No original data is presented in this scoping review.

## Supplementary Materials

The following supporting information can be downloaded at: https://www.mdpi.com/article/10.3390/jcm14113922/s1. File S1. Database Search Strategies—Detailed search strategies used for each electronic database.

## Abbreviations

The following abbreviations are used in this manuscript:

DALY = disability-adjusted life years; RAGT = Robot-assisted gait training; PRISMA-ScR = Preferred Reporting Items for Systematic Reviews and Meta-Analyses Extension for Scoping Reviews; RCT = Randomized controlled trial; EMG = Electromyography; HAL = Hybrid Assistive Limb; GEAR = Gait Exercise Assist Robot; HWA = Honda Walking Assist; QoL = Quality of life; FMA-LE = Fugl-Meyer Lower Extremity Assessment; MAS = Modified Ashworth Scale; RMI = Rivermead Motility Index; 10MWT = 10-Meters Walking Test; 6MWT: 6-Minutes walking test; FAC = Functional Ambulation Category; TUG = Timed Up and Go; FMA = Fugl–Meyer Assessment; EG = experimental group; CG = control group; ADL = activity of daily living; HIT = human–robotic interactive gait training; CPT = conventional physiotherapy; BBS = Berg Balance Scale; ABC = Activities-Specific Balance Confidence Scale; FA = Fractional anisotropy; DTI = diffusion tensor imaging; CSE = cortico-spinal excitability; SMI = cortico-spinal integration; FPEC = frontoparietal effective connectivity; mRS = modified Rankin Scale; BRS = Brunnstrom recovery stage; FIM = Functional Independence Measure; BI = Barthel Index; mBI = modified Barthel Index; VR = Virtual reality; AR = augmented reality; SF-36, SF-8, SF-12 = Short Form Health Survey; EQ-5D = EuroQol-5Dimension; VAS = Visual Analog Scale; GAS = Goal Attainment Scaling; CSQ-8 = Client Satisfaction Questionnaire-8; MMSE = Mini-Mental State Examination; BDI-II = Beck Depression Inventory-II; HRS-D = Hamilton Depression Inventory; NRS = Numeric Pain Rating Scale; BRPE = Borg rating of perceived exertion; mFIS = Modified Fatigue Impact Scale; FSMC = Fatigue Scale for Motor and Cognitive Function; ICF = International Classification of Functioning, Disability, and Health; ADL = Activities of Daily Living; MI-LL = Motricity Index for Lower Limb; WHS = Walking Handicap Scale; AI = Ambulation Index; GCR = Global Rating of Change scale; RMA = Rivermead Motor Assessment Scale; F-MSS = Fugl-Meyer Stroke scale; TMS = Toulouse Motor Scale; TIS = trunk impairment scale; SPPB = short physical performance battery; TIN-B = Tinetti Balance scale; TCT = trunk control test; COP = Center of Pressure; POMA-B = Performance-Oriented Mobility Assessment–balance items; SCP = Scale for Contraversive Pushing; BLS = Burke Lateropulsion Scale; SVV = Subjective Visual Vertical; FAB Frontal Assessment Battery; SCWT = Stroop Colour Word Test; SDMT = Symbol Digit Modalities Test; DCT = Digits Cancellation Test; TMT = Trail Making Test; MMAS = Modified Motor Assessment Scale. ICF = International Classification of Functioning, Disability, and Health; HR = Heart Rate; SIAS = Stroke Impairment Assessment Set; NASA-TLX = NASA Task Load Index; SUS = System Usability Scale; TSQ-WT = Tele-healthcare Satisfaction Questionnaire—Wearable Technology.
