# Supplementary material for: Mapping the Role of Robot-Assisted Gait Training in Post-Stroke Recovery Among Elderly Patients: A Scoping Review
Source: J Clin Med. 2025 Jun 3;14(11):3922. doi: 10.3390/jcm14113922 (PMC12155765; doi:10.3390/jcm14113922)
Supplement: Supplementary file 1 [file jcm-14-03922-s001.zip › jcm-3609038-supplementary.pdf]

SUPPLEMENTARY FILE S1

Database Search Strategies

PubMed, Scopus, Embase, Web of Science, and ClinicalTrials electronic database were searched.

The search formula for each electronic database was:

Pubmed

(stroke) AND (lower limb OR gait) AND (robotic OR exoskeleton OR end-effector OR robot-assisted)

Scopus

(TITLE-ABS-KEY(stroke) AND TITLE-ABS-KEY(gait) OR TITLE-ABS-KEY(lower limb)) AND (TITLE-ABS KEY(robotic) OR TITLE-ABS-KEY(exoskeleton) OR TITLE-ABS-KEY("end-effector"))

Web of Science

([“robotic”] AND [“gait” OR “ambulation”] AND [“rehabilitation” OR “exercise”] AND [“stroke” OR “hemiparesis”]

Embase

'stroke'/mj AND (lower AND 'limb'/mj OR 'gait'/mj) AND (robotic OR 'exoskeleton'/mj OR 'end effector' OR 'robot assisted')

ClinicalTrials.gov

STROKE AND (gait OR lower limb) AND Robotic rehabilitation
